# Tool-Use Training Induces Changes of the Body Schema in the Limb Without Using Tool

**DOI:** 10.3389/fnhum.2019.00454

**Published:** 2019-12-20

**Authors:** Yu Sun, Rixin Tang

**Affiliations:** Department of Psychology, School of Social and Behavioral Sciences, Nanjing University, Nanjing, China

**Keywords:** tool use, body schema, tool embodiment, limb-specific hypothesis, proprioception, plasticity

## Abstract

Previous studies have shown that tool use affects the plasticity of the body schema. In other words, people will perceive the tool as a part of their body, and thus feel like they have “longer limbs” after using tools. However, it is unclear whether tool embodiment could spread to a limb that is not using the tool, and whether other limbs could utilize the proprioception of a limb. In Experiment 1, blindfolded participants were asked to search with a cane (Condition 1) or to walk with a cane (Condition 2). The results in Condition 1 illustrated that the tactile distance perception on the forearm was lengthened after tool use, while other body parts did not significantly change. In Condition 2, the tactile distance perception on the hand and forearm extended significantly after using tools. Additionally, tool-use training even induced an increased perception of the calf that was not using the tool. Possible interference from the difference between walking and standing was excluded in Experiment 2. These results demonstrate that the proprioception information of one limb could be exploited by another limb to extend the body schema even though that limb was not using a tool. It was also observed that the effect of direction was task-dependent in the tactile perception task.

## Introduction

Tool use contributes to human survival by allowing humans to reach inaccessible spaces and protect their bodies from harm. Many studies have revealed that participants tend to perceive tools as part of their own bodies after tool use, their perception of their body parts is extended, and their body schema is changed, which is also known as the phenomenon of tool embodiment (e.g., Iriki et al., 1996; Cardinali et al., 2009, 2011; Sposito et al., 2012). Humans use tools more efficiently and accurately when it is incorporated into the body schema, allowing the brain to control it just like other body parts (Cardinali et al., 2016a). Perhaps tool embodiment is more crucial for individuals who are more dependent on the tools. For example, amputees could use prostheses to perform movements with greater flexibility, and blind people could use canes to explore the spaces around them more efficiently.

Previous studies emphasized training a specific limb with the tool then testing the body schema of the same limb. Nonetheless, it is still unclear whether the phenomenon of tool embodiment is based on the specific limb or the general procedure for all limbs. Some studies have discovered that using tools with the hand only changed the body schema of the forearm, but did not change the body schema of the foot (Jovanov et al., 2015) or the cheek (Miller et al., 2017a). Additionally, Miller et al. (2014) found that only the limb that is similar to the morphology of a tool could be modified by tool use. However, in previous studies, the participants used the tool with their hands, and the tool accordingly gave functional benefits to the hands by expanding the space that the hands can reach. Therefore, it was reasonable that the body schema of other limbs failed to change. It is still unclear whether the body schema of limbs that do not use a tool would change when tools give functional benefits to that limb. If the body schema of the limb not using a tool changed, then the tool embodiment is general to all limbs. Otherwise, tool embodiment is limb-specific.

Sensory input from multiple modalities–such as vision, proprioception, and tactile sensation—played an important role in the incorporation of tools into the body schema (Miller et al., 2015; Cardinali et al., 2016b; Martel et al., 2019). The proprioception provides information on perceived position and movement of limbs and the body without visual feedback (Gilman, 2002). It was considered that the proprioception was necessary (Cardinali et al., 2016b) and sufficient (de Vignemont et al., 2005; Martel et al., 2019) to induce the changes of the body schema. Furthermore, the proprioceptive input may be dominant to the body representation even compared to vision (Shenton et al., 2004). Several neurophysiology and lesion studies have also suggested that the body schema is predominantly based on proprioception (Head and Holmes, 1911; Paillard, 1999; Gallagher, 2005). It is clear that visual information could be used to control all limbs, but proprioception is fixed to the specific limb. Blindfolded and blind participants could adapt to the new force environment on the basis of proprioception (DiZio and Lackner, 2000). The proprioceptive information from one limb arrives at the primary somatosensory cortex (SI) from the thalamus (Kaas et al., 1979). It was then possible for the proprioceptive information of one limb stored in the SI to be used by other limbs, so as to drive dynamic adaptation when walking blindly. It should be noted that the visual feedback was usually available in most of the tool studies previously conducted, in which the proprioception information did not make a significant difference in the tasks. However, when the visual feedback was absent, the limb not using the tool became dependent upon the proprioceptive information of the limb using the tool to program its movement. It is interesting to study whether the proprioception of the limb using the tool could be exploited by other limbs not using the tool, as well as whether the proprioceptive information related to tool could induce changes in the body schema of the limb not using the tool.

In this study, two experiments were conducted to investigate whether tool embodiment could spread to the limb not using the tool and whether proprioception is general to all limbs or specific to one limb. In Experiment 1, blindfolded participants were instructed to search for the target object with a cane (Condition 1) or to walk with a cane (Condition 2). They performed a tactile distance perception task before and after the training to investigate if the body schema of their hands, forearms, feet, or calves had changed. It was expected that limbs that experienced a change in body schema would be different between Condition 1 and Condition 2, even though the same limb used the same tools in both experiments. Possible interference from the difference between walking and standing was studied in Experiment 2.

## Experiment 1

### Participants

Fifty-six participants (31 females; mean age ± SD: 21.71 ± 2.10; ranging from 18 to 28 years of age) took part in Condition 1 and Condition 2. All participants were right-handed and had normal or corrected normal vision. They all received monetary compensation for their participation. All participants gave written and informed consent to participate in the study, which was approved by the Ethical Committee of the Department of Psychology, Nanjing University. These participants also attended Experiment 2 on different days.

### Apparatus and Procedures

**Figure 1:**
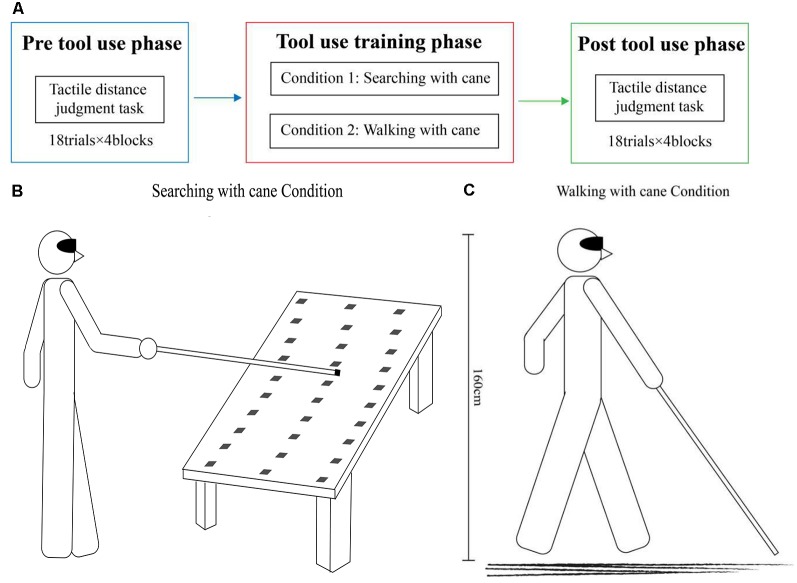
Experimental design. **(A)** The entire experiment procedure was divided into three phases: pre-tool-use phase, tool-use-training phase (20 min), and post-tool-use phase. In the pre- and post-tool-use phases, participants performed a tactile distance perception task. The three types of tactile stimuli were administered into their hands, forearms, feet, and calves in separate blocks. Every block included 18 trials.** (B)** In the searching with cane condition, participants were instructed to use a cane to find the target object, which would be randomly placed in one of 30 possible positions. The longitudinal distance covered a range of 100–160 cm from the body of the participant to the target object, and the transversal distance covered a range of 0–90 cm.** (C)** In the walking with cane condition, participants were instructed to walk with a cane for 20 min.

As shown in Figure 1A, all participants were required to perform two experimental conditions and both experimental conditions were composed of three phases: a pre- and post-tool-use session (18 trials × 4 blocks each), during which participants were instructed to perform a tactile distance perception task, separated by a tool-use training session. The two experimental conditions differed only in the tool-use training session.

The specific experimental procedures were as follows. At the beginning of the experiment, participants would be instructed to put on a blindfold, and this condition of having no vision would last the entire experiment. Tactile distance perception task would then be performed on the hands, forearms, feet, and calves of the participants. During this task, participants comfortably sat on a chair and placed their right hand on the table, while also placing their right leg on the other chair. Every time before starting the tactile distance perception task, participants would practice at least five times to ensure that they could complete the task. This was interspersed with a tool-use training task between two tactile distance perception tasks. During the training task, participants were asked to search for the object with the cane (Condition 1) and walk with the cane (Condition 2) on two different days.

#### Tactile Distance Perception Task

The tactile distance perception task is an implicit and sensitive task for measuring the plasticity of the body schema (Taylor-Clarke et al., 2004; de Vignemont et al., 2005; Longo and Haggard, 2011; Tajadura-Jiménez et al., 2012). In this task from previous studies, participants were instructed to verbally report which body part (target body part, e.g., forearm; reference body part, e.g., forehead) that was touched was perceived longer (Canzoneri et al., 2013; Miller et al., 2014). In the version of the tactile distance perception task, participants made verbal estimations of the distance between two tactile points manually applied to the target body part (Longo and Sadibolova, 2013; Miller et al., 2017a). In the present study, the tactile distance perception task was adapted from Longo and Sadibolova (2013) and Miller et al. (2017a). Tactile points were administered manually and longitudinally (from wrist to knuckles) to four target body parts (the dorsal surface of the right hand, right forearm, right foot, and right calf) with a stainless steel digital caliper in separate blocks. There were three types of tactile distances (separated by 20, 30, or 40 mm), and every tactile distance was administered six times for a total of 18 trials in every block. The order was random. The tactile stimuli administered to the body parts of the participants lasted for approximately 1 s, and then the participants verbally reported the estimated distance in millimeters. Participants were blindfolded throughout the procedure. There was no limit to the time that participants made their verbal reports.

#### Tool-Use Training

In the condition of searching with a cane (see Figure 1B), the tool-use training was adapted from Serino et al. (2007) and Canzoneri et al. (2013). The tool was a 120 cm aluminum alloy cane with a diameter of 13 mm. The blindfolded participants were required to find a 4 × 4 × 8 cm wooden target object randomly placed in one of 30 different locations on the table, and to knock the front, top left, and right sides of the target with the cane. There were three possible longitudinal distances from the body (100 cm, 130 cm, and 160 cm), and 10 transversal positions covering a space ranging from 0 to 90 cm. All participants stood at a fixed starting position and used the cane with their right hands. At the beginning of the training, the experimenter placed the target object randomly in one of 30 possible locations on the table, avoiding making any sounds that could give the participants a hint about the exact location of the object. Participants were then instructed to explore the space in front of them with the cane, imitating the movements that blind people use with a cane until they found the object. Once the participants found the target, they knocked it over. Then the experimenter removed the first object and placed another one on the table. There were no time constraints for how quickly the participants had to find the target object.

In the condition of walking with the cane (see Figure 1C), the tool-use training required blindfolded participants to walk with the cane in a similar way that a blind person would navigate himself or herself. In the 20 min of training, participants needed to explore the space around them and find their way with the help of the tool. The tool was the same as that used in Condition 1. In this experimental condition, the experimenter would accompany participants in case of accidents but avoided making any sounds that could interfere with the participants. To reduce auditory interference, rubber was attached to the bottom of the cane to lessen the sound. Additionally, participants were asked to use the cane in a similar way in the two tasks to reduce the effect of the sound caused by different usages of the tool.

### Results

The normality of data was checked and most of the data conformed to normal distribution. For the data that deviated from normality, either a repeated ANOVA was conducted after replacing the outliers with means or a non-parametric test was done when the outliers did not cause the deviation from normality. As for the data satisfying the normal distribution, three 2 (Condition: searching with cane, walking with cane) × 2 (Phase: pre, post) × 3 (Tactile distance: 20 mm, 30 mm, 40 mm) repeated measure ANOVAs were separately conducted for the verbal estimated tactile distances of three limbs. The current study concerned the different modulations under different tool-use training tasks, where the interactions between factors Condition and Phase was crucial to the goals of the present study.

**Figure 2:**
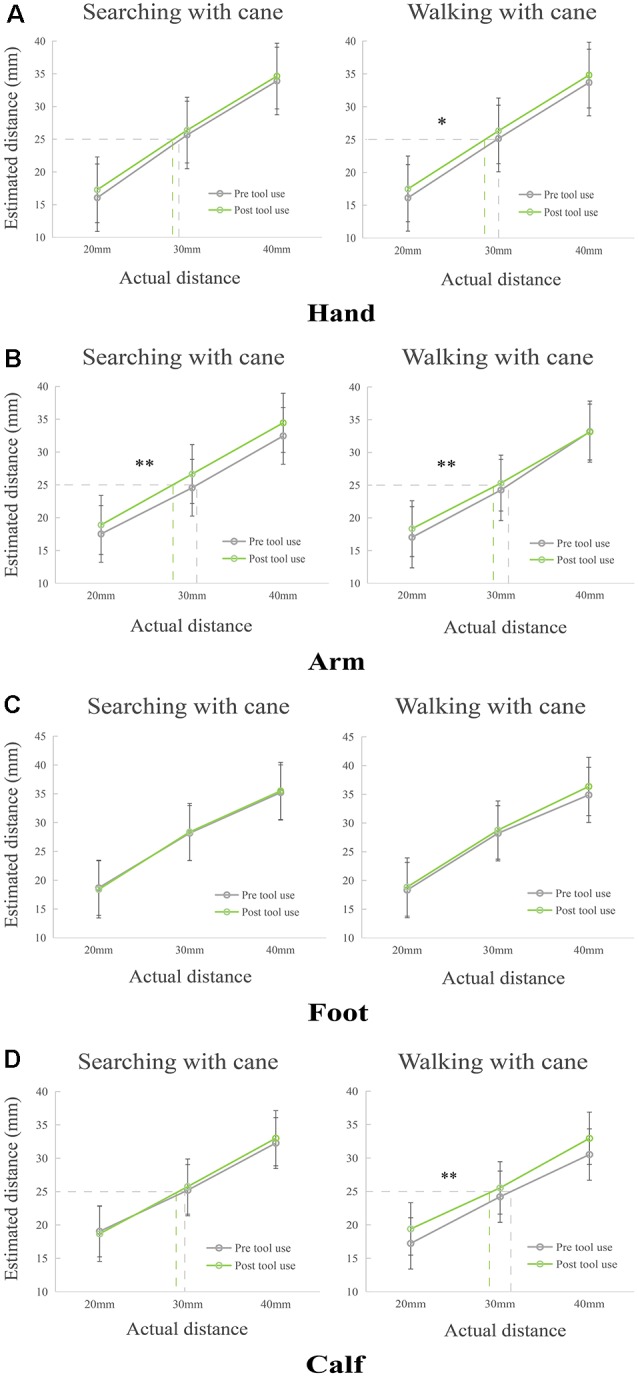
Modulations of perceived distance on different limbs in Condition 1 and Condition 2. **(A)** The perceived distance of hand extended significantly in the task of walking with a cane, but it remained unaltered in the searching with cane condition. **(B)** The estimated tactile distance of the forearm significantly increased after tool use training of searching with cane and walking with cane. **(C)** The perceived distance of the foot in both conditions. **(D)** The perception of the calf extended significantly after walking with a cane, but it remained unchanged in the task of searching with cane. *p < 0.05, **p < 0.01.

For the analysis of hands (see Figure 2A), two non-parametric tests between pre- and post-tool use were conducted because Shapiro–Wilk tests signaled that the data deviated from normality. The non-parametric test of walking with cane condition showed that there was a significantly increased perception after using tools (*p* = 0.014). Meanwhile, a marginally significant increase between pre- and post-tool use in the condition of searching with a cane (*p* = 0.066) was found. Additionally, significant differences among three tactile distances in both conditions were found (*p*_max_ < 0.001), demonstrating that participants indeed increased their estimations as the actual distance increased.

The repeated ANOVA of arms (see Figure 2B) showed significant main effects of Phase (*F*_(1,55)_ = 14.135, *p* < 0.001, *η*_p_ = 0.204) and Tactile distance (*F*_(2,110)_ = 500.829, *p* < 0.001, *η*_p_ = 0.901). The main effect of Phase showed an increased perception after tool use in both conditions. No other main effects or interactions were found (*F*_(1,55)max_ = 2.518, *p*_min_ = 0.118, *η*_p_ = 0.044).

For the repeated ANOVA of feet (see Figure 2C), a significant main effect of Tactile distance (*F*_(2,110)_ = 970.033, *p* < 0.001, *η*_p_ = 0.946) was found. There were no other main effects or interactions (*F*_(1, 55)max_ = 2.308, *p*_min_ = 0.134, *η*_p_ = 0.040).

Finally, for the analysis of calves, a repeated ANOVA was conducted. The results (see Figure 2D) showed that there was a significant interaction between factors Condition and Phase (*F*_(1,55)_ = 7.223, *p* = 0.010, *η*_p_ = 0.116). Two significant main effects were found for Tactile distance (*F*_(2,110)_ = 459.361, *p* < 0.001, *η*_p_ = 0.893), and Phase (*F*_(1,55)_ = 13.729, *p* < 0.001, *η*_p_ = 0.200). Simple effect on the calf showed that perceived tactile distance of post tool use was longer than that of pre tool use in the condition of walking with cane (*p* < 0.001), but it did not change after tool use in the condition of searching with cane (*p* = 0.468). No other main effects or interactions were observed (*F*_(1,55)max_ = 2.849, *p*_min_ = 0.097, *η*_p_ = 0.049).

## Experiment 2

A previous study has suggested that the peripersonal space expands in the case of walking rather than standing still (Noel et al., 2015). The body schema expansion of calf found in Experiment 1 might result from the walking movement. In Experiment 2, whether the walking movement would affect the body schema of the calf was particularly emphasized.

### Methods

The participants were the same as that of Experiment 1. The apparatus and procedures were consistent with Condition 2 except for the walking phase. In the walking phase of Experiment 2, participants were instructed to walk without the cane.

### Results

**Figure 3:**
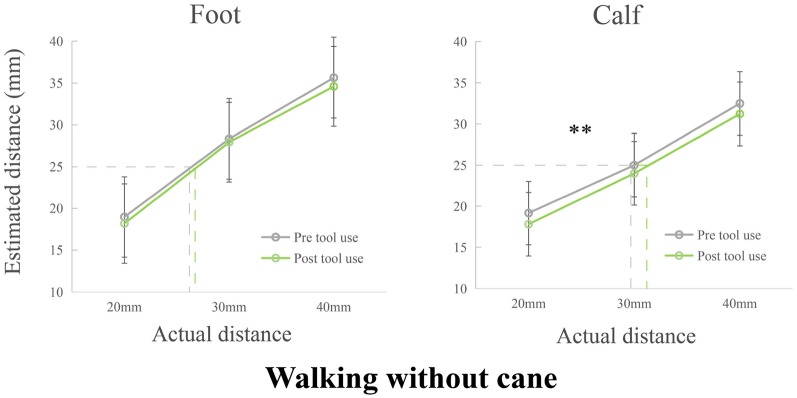
Modulations of the perceived distance of the foot and calf in Experiment 2. The perceived distance remained unchanged on the foot. However, the perceived distance of post tool use was significantly shorter than that of pre tool use on the calf. **p < 0.01.

The tests of normality showed that all data including difference scores was normally distributed after replacing an outlier with mean. Two 2 (Phase: pre, post) × 3 (Tactile distance: 20 mm, 30 mm, 40 mm) repeated measure ANOVAs were separately performed with verbal distance estimations on the foot and calf (see Figure 3). The repeated ANOVA of foot showed a significant main effect of Tactile distance (*F*_(2,110)_ = 493.170, *p* < 0.001, *η*_p_ = 0.900). No other main effect (*F*_(1,55)_ = 3.289, *p* = 0.075, *η*_p_ = 0.056) or interaction (*F*_(2,110)_ = 0.571, *p* = 0.567, *η*_p_ = 0.010) was found. The repeated ANOVA of calf implied that there were significant main effects of Phase (*F*_(1,55)_ = 9.023, *p* = 0.004, *η*_p_ = 0.141) and Tactile distance (*F*_(2,110)_ = 269.746, *p* < 0.001, *η*_p_ = 0.831). The main effect of Phase showed a decreased perception after walking without cane. There were no interaction (*F*_(2,110)_ = 0.180, *p* = 0.836, *η*_p_ = 0.003).

**Figure 4:**
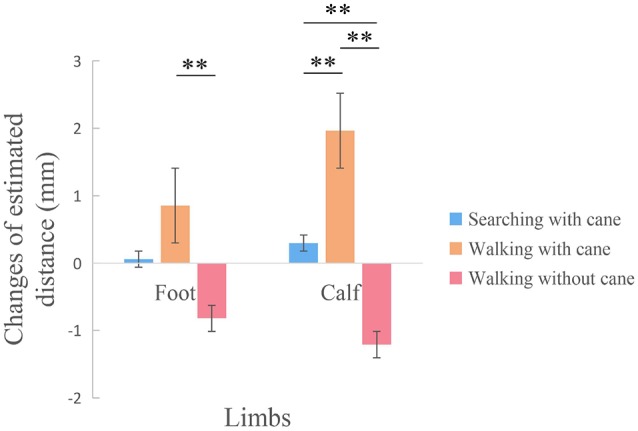
The comparison among three conditions with difference scores between pre and post tool use. There was a significant difference on the foot between walking with cane condition and walking without cane condition. Additionally, there were significant differences among the three conditions on the calf. **p < 0.01.

The effects of tool use in three different conditions on the calf and foot were calculated with difference scores (the estimated distance of post tool use—the estimated distance of pre tool use). Two 3 (Condition: searching with a cane, walking with cane, walking without a cane) × 3 (Tactile distance: 20 mm, 30 mm, 40 mm) repeated measures ANOVAs were separately conducted for the foot and calf (see Figure 4). The repeated ANOVA of the foot suggested that there was a significant main effect of Condition (*F*_(2,110)_ = 4.440, *p* = 0.014, *η*_p_ = 0.075). The *post hoc* test showed that the estimated distance of walking with cane condition was significantly longer than that of walking without cane (*p* = 0.003), but there were no significant differences between searching with cane and walking with cane (*p* = 0.185), or between searching with cane and walking without cane (*p* = 0.119). There were no other main effect (*F*_(2,110)_ = 1.190, *p* = 0.308, *η*_p_ = 0.021) or interaction (*F*_(4,220)_ = 0.860, *p*_min_ = 0.489, *η*_p_ = 0.015) on the foot. Additionally, a significant main effect of Condition was observed on the calf (*F*_(2,110)_ = 16.658, *p* < 0.001, *η*_p_ = 0.232). Specifically, the *post hoc* test showed that there were significant differences between walking with a cane and searching with a cane (*p* = 0.004), significant differences between walking with cane and walking without cane (*p* < 0.001), and significant differences between searching with cane and walking without cane (*p* = 0.009). No other significant main effect (*F*_(2,110)_ = 0.584, *p* = 0.559, *η*_p_ = 0.011) or interaction (*F*_(4,220)_ = 1.151, *p* = 0.331, *η*_p_ = 0.021) on the calf were found.

## Discussion

In this study, the change in body schema of the limb not using the tool that received functional benefits from the tool was investigated, and whether proprioception was general to all limbs or specific to one limb was also analyzed. Two experiments were conducted to answer these questions. Blindfolded participants were instructed to search for the target object with a cane (Condition 1) or walk with a cane (Condition 2). In Condition 1, it was revealed that the perceived tactile distance applied to the forearm was significantly extended after tool use, and the perception of the hand showed a marginally significant increase after tool use, while the body schema of other limbs remained unaltered. In Condition 2, the results showed that the tactile distance perception on the hand and forearm extended significantly after using tools. Additionally, tool-use training even induced an increased perception of the calf that was not using the tool. Furthermore, in Experiment 2, the potential interference of walking was excluded by instructing participants to walk blindfolded without the cane.

As was predicted, the body schema of the forearm changed after using the cane to touch the target object and to walk, which was consistent with previous studies (e.g., Iriki et al., 1996; Cardinali et al., 2009, 2011; Sposito et al., 2012). Even though the visual information was unavailable, it was still revealed that participants exhibited extended forearms after tool use in Experiment 1, indicating that only proprioception could change body schema (de Vignemont et al., 2005; Martel et al., 2019). Additionally, the body schema of the hand showed a significantly increased perception in Condition 2 and a marginally significant increased perception in Condition 1, which was inconsistent with the studies conducted by Miller et al. (2014). This was perhaps due to differences in the experimental tasks. In the task of Miller et al. (2014), participants squeezed a vertical handle to control pincers at the tooltip. The movement of the hand either curling up or squeezing potentially caused the shrinkage of perception on the hand. It may also cause the separation of the hand and forearm due to their different methods of movement. In the present study, it is likely that the dorsal of the hand and forearm were a whole because of the same movement used between them. Moreover, the morphology of the dorsal of the hand was similar to that of the forearm and tool to some extent. Therefore, the perception of the hands produced the same changes as that of the forearms.

In Condition 2, when the cane was used by the hand to assist with walking, the body schema of the hand and forearm changed. Importantly, the body schema of the calf that was not using the tool experienced a change. The different modulations on the calves between Condition 1 and Condition 2 suggest that different functions of tool use caused different changes in the body schema of limbs. This result is consistent with previous studies emphasizing the functionality of tools. For example, the functional length of the tool was more important to body representation than physical length (Farnè et al., 2005; Sposito et al., 2012). Additionally, Reed et al. (2010) found that the acceleration of target stimulus recognition only occurred at the functional side of the tool and that there was no effect on the other side.

In the present study, the functionality of tools even altered the body schema of the limb not using the tool. One potential explanation for this is that blindfolded participants in the walking with cane condition paid more attention to the lower limbs than that of searching with cane condition. The tactile perception then became larger after training. However, it is unlikely to happen because the visual attention is usually prioritized for the space near the distal of limbs (Makin et al., 2007; Brozzoli et al., 2011; Gentile et al., 2011) or the tip of tools (Maravita et al., 2001; Farnè et al., 2005; Kao and Goodale, 2009; Reed et al., 2010), causing the faster detection of visual targets. Therefore, it should have been determined that the body schema of the foot in Condition 2 was affected after tool use. It should be noted that the body schema of the foot was not affected in the present study. A possible reason for this is that the activation of leg muscles is fundamental to the control of human walking (Franz and Kram, 2012) rather than the foot muscles. Moreover, the vertical positional relationship between foot and calf while walking perhaps induced that the morphology of cane was more similar to the calf. Thus, the calf-shaped cane only changed the body schema of the calf, but not that of the foot (Miller et al., 2014; Cardinali et al., 2016a).

Another possibility is that the sensorimotor representation of the calf was activated when participants used the proprioceptive information obtained from the tooltip to program its movement (see Miller et al., 2017b). The triangle formed among the cane, the calf, and the ground appeared to be an extension of the reachable area of the calf. In contrast, blindfolded participants would not dare to walk without the cane due to the fear of falling, thus showing a slower walking speed (Hallemans et al., 2010). The mental state of not daring to walk possibly induced a perception that the reachable area of the calf was shrinking, further leading to a decreased perception of tactile distance applied to the calf. The proprioception information obtained from the tooltip includes the perceived length of the tools (Solomon et al., 1989), the size of object that came into contact with the tool (Turvey et al., 1998), as well as the relation between self and the surrounding environment (Harrison and Turvey, 2010; Turvey and Carello, 2011). In the present study, proprioception information of tool use was exploited to manage walking blindly, which is similar with the study conducted by Harrison and Turvey (2010), in which the blindfolded participants could have place learning by walking, stepping, and cane probing. Moreover, motor programming might play a role in the plasticity of body schema since the motor imagery (Baccarini et al., 2014) and visual illusion (Miller et al., 2017b) of tool use can change the tactile perception on the stationary arms. This also suggests that the proprioception fixed to one limb can be used by another limb and change their body schema, reflecting the common representation of proprioception in the brain.

It should also be noted that an increased perception in the tactile distance within the current study was longitudinal and not transversal. The increased perception in the longitudinal orientation was interpreted as an increase in the represented size of the body part (de Vignemont et al., 2005; Tajadura-Jiménez et al., 2012). However, this effect of direction was different from the studies conducted by Canzoneri et al. (2013) and Miller et al. (2014), which were transversal. Romano et al. (2019) suggested that shoulder- or wrist- training induced different changes in body representation. The training of previous studies involved arm retraction (Canzoneri et al., 2013) or bending (Miller et al., 2014), which depended more on the proximal part rather than the distal part. In the present study, the arms of the participants stretched to use the cane to search or navigate in the tasks, which were more dependent on the distal part. This indicates the current effect is task-dependent.

Overall, the current study shows that different goals of tool use caused the different changes of the body schema. Importantly, tool use could induce the body schema changes of the limb even though that limb was not using a tool. Furthermore, the direct effect of tactile distance perception tasks might be task-dependent. Finally, the present study also tested and verified that tool use could cause the changes of body schema on the sole basis of proprioception.

## Data Availability Statement

The datasets generated for this study are available on request to the corresponding author.

## Ethics Statement

This study was carried out in accordance with the recommendations of the Ethical Committee, Nanjing University. All participants gave written and informed consent to participate in the study. The protocol was approved by the Ethical Committee of the Department of Psychology, Nanjing University.

## Author Contributions

RT and YS designed the experiment, discussed the results and wrote the article. YS conducted the experiment and analyzed the data under the supervision of RT.

## Conflict of Interest

The authors declare that the research was conducted in the absence of any commercial or financial relationships that could be construed as a potential conflict of interest.
